# Mammographic density and risk of breast cancer by tumor characteristics: a case-control study

**DOI:** 10.1186/s12885-017-3871-7

**Published:** 2017-12-16

**Authors:** Kavitha Krishnan, Laura Baglietto, Jennifer Stone, Catriona McLean, Melissa C. Southey, Dallas R. English, Graham G. Giles, John L. Hopper

**Affiliations:** 1 Centre for Epidemiology and Biostatistics, Melbourne School of Population and Global Health, University of Melbourne, Level 3, 207 Bouverie Street, Carlton, VIC 3053 Australia; 2 Cancer Epidemiology Centre, Cancer Council Victoria, Melbourne, Australia; 3 Université Paris-Saclay, Univ. Paris-Sud, UVSQ, CESP, INSERM, Villejuif, France; 4 Gustave Roussy, F-94805 Villejuif, France; 5 Centre for Genetic Origins of Health and Disease, University of Western Australia, Perth, Australia; 6 The Alfred Hospital, Melbourne, Australia; 7 Genetic Epidemiology Laboratory, Department of Pathology, University of Melbourne, Melbourne, Australia; 8 Department of Epidemiology and Preventive Medicine, Monash University, Melbourne, Australia; 9 Seoul Department of Epidemiology, School of Public Health, Seoul National University, Seoul, South Korea; 10 Institute of Health and Environment, Seoul National University, Seoul, South Korea

**Keywords:** Mammographic density, Breast cancer, Detection mode, Tumor characteristics

## Abstract

**Background:**

In a previous paper, we had assumed that the risk of screen-detected breast cancer mostly reflects inherent risk, and the risk of whether a breast cancer is interval versus screen-detected mostly reflects risk of masking. We found that inherent risk was predicted by body mass index (BMI) and dense area (DA) or percent dense area (PDA), but not by non-dense area (NDA). Masking, however, was best predicted by PDA but not BMI. In this study, we aimed to investigate if these associations vary by tumor characteristics and mode of detection.

**Methods:**

We conducted a case-control study nested within the Melbourne Collaborative Cohort Study of 244 screen-detected cases matched to 700 controls and 148 interval cases matched to 446 controls. DA, NDA and PDA were measured using the Cumulus software. Tumor characteristics included size, grade, lymph node involvement, and ER, PR, and HER2 status. Conditional and unconditional logistic regression were applied as appropriate to estimate the Odds per Adjusted Standard Deviation (OPERA) adjusted for age and BMI, allowing the association with BMI to be a function of age at diagnosis.

**Results:**

For screen-detected cancer, both DA and PDA were associated to an increased risk of tumors of large size (OPERA ~ 1.6) and positive lymph node involvement (OPERA ~ 1.8); no association was observed for BMI and NDA. For risk of interval versus screen-detected breast cancer, the association with risk for any of the three mammographic measures did not vary by tumor characteristics; an association was observed for BMI for positive lymph nodes (OPERA ~ 0.6). No associations were observed for tumor grade and ER, PR and HER2 status of tumor.

**Conclusions:**

Both DA and PDA were predictors of inherent risk of larger breast tumors and positive nodal status, whereas for each of the three mammographic density measures the association with risk of masking did not vary by tumor characteristics. This might raise the hypothesis that the risk of breast tumours with poorer prognosis, such as larger and node positive tumours, is intrinsically associated with increased mammographic density and not through delay of diagnosis due to masking.

## Background

Mammographic density (MD) is a risk factor for breast cancer [1–5] and is also known to play a role in the masking of the tumor [1, 2, 5]. What is unclear is whether the influences of MD on inherent risk and masking vary by tumor characteristics. Knowledge on this might aid to understand the aetiology of breast cancer. Specifically, understanding if the dense tissues of the breast give rise to breast tumors of a specific kind might aid in understanding the biological mechanisms involved in the development of breast tumors. Understanding if masking varies by tumor characteristics might highlight the difference in the biology of the various tumors.

In a previous paper [6], we postulated that the risk of screen-detected breast cancer is mostly influenced by inherent risk, while risk of interval breast cancer is due to a combination of inherent risk and risk of masking. Therefore, given a woman participating in a screening program is diagnosed with breast cancer, the factors associated to the likelihood of having a screen-detected versus an interval cancer will mostly be those that influence risk of masking. We reported in the paper that inherent risk was predicted by body mass index (BMI) and dense area (DA) or percent dense area (PDA), but not by non-dense area (NDA), whereas masking was best predicted by PDA but not BMI [6].

Very few studies have analysed the association between MD and risk of breast cancer by tumor characteristics, separately for each detection mode [7–10]. Of these one study did not adjust for BMI [9], the rest did not allow for the association between BMI and risk to vary by age [7, 8, 10] and none of them had investigated the concurrent associations of dense area (DA), percent dense area (PDA) and non-dense area (NDA).

For screen-detected cancer, studies have observed that higher density was associated with increased risk of larger tumors [7, 10] and nodal involvement [7] after adjusting for BMI. Results for interval cancer are more varied. One study observed a negative association between density and histologic grade, differentiation and mitotic index after adjusting for BMI but there was no statistically significant difference in the risk estimates between screen-detected and interval cases [10]. This might not be surprising as 66% of the interval cases were true interval cases thus, the risk of interval cancer would most likely reflect inherent risk similar to risk of screen-detected cancer as postulated in our paper. Another study had combined interval cases with clinically detected cases (i.e. women with breast symptoms referred to for mammography) and reported density to be positively associated for oestrogen (ER)- and triple-negative tumors [8] and larger tumors [7] after adjusting for BMI.

Here we have used the same case-control study nested within the Melbourne Collaborative Cohort Study (MCCS) analysed before [6], to investigate if the association between DA, PDA and NDA and inherent risk of breast cancer, and the risk of masking vary by tumor characteristics, specifically size, grade, lymph node involvement, and ER, progesterone (PR), and human epidermal growth factor receptor 2 (HER2) status.

## Methods

The MCCS is a prospective cohort study, which started recruiting participants from the Melbourne metropolitan area between 1990 and 1994. At study entry there were 41,514 participants (including 24,469 women) aged between 27 and 76 years. A nested case-control study was designed based on the subset of MCCS women who had been identified to have attended BreastScreen Victoria, a population-based screening program, through a record linkage conducted in 2009 (20,444 women). Cases were women who subsequently had a first diagnosis of invasive adenocarcinoma of the breast (International Classification of Diseases for Oncology codes C50.0–C50.9). Four controls were matched to each case by year of birth, year of entry into the MCCS and country of origin. The mammogram with craniocaudal view and closest to study entry was chosen for measurement. Screen-detected cases were identified at BreastScreen Victoria. Cases diagnosed within 2 years of a negative screen at BreastScreen Victoria were defined as interval cases. There were 244 screen-detected cases matched to 700 controls and 148 interval cases matched to 446 controls. Further details about the nested case-control study have been published elsewhere [6, 11, 12].

### Tumor characteristics

The Victorian Cancer Registry (VCR) reviewed the pathology reports and classified the cancers according to tumor size, tumor grade, lymph node involvement, and ER, PR, and HER2 status. The original diagnostic tumor slides were retrieved for 85% of the cases from pathology laboratories and reviewed by a single pathologist (C. McLean) who assessed ER, PR, and HER2 status using immunohistochemistry techniques [13]. ER and PR tumors were categorized as positive if ≥ 1% of the nuclei were stained and/or the intensity of staining was weak, moderate, or strong and negative otherwise; HER2 tumors were categorized as positive if > 10% of the nuclei were stained and the intensity of staining was weak, moderate, or strong and negative otherwise. The agreements between the ER, PR, and HER2 status assessed by immunohistochemistry and the records held by the VCR were 91%, 70%, and 72%, respectively (for ER, κ = 0.56, *P* < 0.0001; for PR, κ = 0.30, *P* < 0.0001; for HER2, κ = 0.32, *P* < 0.0001). Given the good agreement between the ER, PR, and HER2 data, when archival tumor tissue was not available, ER, PR, and HER2 status was assigned according to the histopathology reports held at the VCR.

### Statistical analyses

Associations between the mammographic measures and risk were estimated in terms of odds per adjusted standard deviation (OPERA) according to models with different combinations of the variables, DA, PDA, NDA and BMI, as mentioned in our previous publication [6]. Further details about OPERA have been published elsewhere [14–16].

Firstly, by applying the Box-Cox method for transforming variables to the mammographic measures of the control group, DA, PDA and NDA were transformed to (DA^0.2^–1)/0.2, (PDA^0.2^–1)/0.2 and (NDA^0.5^–1)/0.5, respectively. Linear regression was applied on each transformed mammographic measure, adjusting for age at mammogram, BMI (standardized according to the controls) and all the matching variables, and the residuals were standardized to have zero mean and unit variance. Conditional logistic regression of case-control status was then applied separately for screen-detected and interval cancers and for each tumour characteristic. The type-specific OPERA estimates were obtained by fitting an interaction term between the standardised residuals and a set-specific variable equal to the tumour type of the matching case. Heterogeneity by tumor characteristics was assessed using likelihood ratio test. Age at mammogram was fitted as a potential confounder.

For the models that included BMI measured at the MCCS study entry, we fitted an interaction between BMI (standardized based on the controls) and reference age (age at diagnosis for the case and for her matched controls) and its significance was assessed using the likelihood ratio test. We have reported the risk estimates for BMI at ages 50 and 70 to show the predicted risks corresponding to the pre- and postmenopausal age groups.

For the analyses of interval versus a screen-detected breast cancers, unconditional logistic regression was applied only to cases adjusted for age at mammogram. Association between BMI and risk of interval versus a screen-detected breast cancer did not depended on age at diagnosis.

The Bayesian information criterion (BIC) and the area under the receiver operating characteristic curve (AUC) were used to test for relative goodness of fit. Differences between AUCs were tested using the De Long’s tests [17].

Sensitivity analyses were conducted in which we further adjusted for potential confounders: BMI at age 18–21 years; age at menarche; parity and lactation; menopausal status; HRT use; OC use; alcohol consumption and energy intake; and the matching variables (country of birth, year of birth, year of cohort entry and reference age) using unconditional logistic regression. We further adjusted for family history of breast cancer. A sensitivity analysis was also conducted by excluding cases diagnosed within 2 years from the mammogram, and their matching controls.

A more detailed explanation of the method used to derive OPERA has been given in our previous publication [6].

Statistical analyses were performed using Stata 12.1 (Stata Corporation, College Station, TX). Two-sided *P* < 0.05 was considered to be nominally statistically significant.

## Results

As shown in Table 1, screen-detected cases were on average about 2 to 3 years older than interval cases at diagnosis (65 years vs 62 years, *P* < 0.001), at study entry when covariates were measured (56 years vs 54 years, *P* = 0.01), and at the mammogram closest to study entry (59 years vs 57 years, *P* < 0.01). Interval cases had on average greater DA and PDA and lesser total breast area and NDA than screen-detected cases (*P* < 0.01). There was no significant difference in BMI and all the other confounders except for menopausal status and alcohol consumption between the two types of cases. Within the screen-detected cases there were a higher percentage of menopausal women (*P* = 0.02) and lower percentage of alcohol consumers (*P* < 0.01) at cohort entry than the interval cases.

**Table 1:** Characteristics of study participants

	Screen-detected cases	Controls	Interval cases	Controls
	(*N* = 244)	(*N* = 700)	(*N* = 148)	(*N* = 446)
	Mean (SD)	Mean (SD)	Mean (SD)	Mean (SD)
Age at baseline, years	56 (8)	56 (8)	54 (8)	54 (8)
Age at mammogram, years	59 (7)	59 (7)	57 (7)	57 (7)
Age at diagnosis, years	65 (7)		62 (7)	
Time between age at mammogram and reference age, years	6 (3)	6 (3)	5 (3)	5 (4)
Total energy intake, MJ/day	8.4 (2.9)	8.6 (3.5)	8.7 (3.2)	8.7 (3.1)
BMI, kg/m2				
All women	27.5 (4.9)	26.7 (4.9)	26.7 (5.3)	26.5 (4.8)
Premenopausal	27.2 (5.7)	26.2 (5.0)	25.9 (4.9)	26.1 (4.6)
Postmenopausal	27.6 (4.6)	27.0 (4.9)	27.2 (5.5)	26.7 (5.0)
BMI at age 18–21 years, kg/m2	21.5 (2.9)	21.5 (2.9)	21.4 (2.5)	21.5 (2.8)
Breast				
Total area, cm2	143.7 (60.9)	137.8 (57.6)	125.0 (56.9)	133.4 (60.3)
Non-dense area, cm2	124.2 (62.1)	120.9 (58.8)	96.5 (55.6)	115.3 (60.6)
Dense area, cm2	19.6 (21.7)	16.8 (20.6)	28.5 (24.5)	18.1 (19.4)
Percent mammographic density, %	15.5 (15.7)	14.0 (15.3)	25.2 (17.9)	15.4 (14.7)
Country of birth	N (%)	N (%)	N (%)	N (%)
Anglo Saxon/ Other	204 (84)	583 (83)	123 (83)	371 (83)
Italy	25 (10)	74 (11)	13 (9)	40 (9)
Greece	15 (6)	43 (6)	12 (8)	35 (8)
Age at menarche, years				
< 12	47 (19)	125 (18)	31 (21)	75 (17)
12	52 (21)	145 (21)	25 (17)	86 (19)
13	55 (23)	169 (24)	36 (24)	111 (25)
14+	88 (36)	261 (37)	56 (38)	174 (39)
Parity and lactation				
Nulliparous	38 (16)	84 (12)	25 (17)	62 (14)
Parous, never lactated	10 (4)	61 (9)	12 (8)	34 (8)
Parous, lactated	190 (78)	542 (77)	108 (73)	343 (77)
Menopausal status				
Premenopausal	69 (28)	195 (28)	59 (40)	170 (38)
Postmenopausal	174 (71)	504 (72)	89 (60)	275 (62)
Hormone replacement therapy use				
Never	169 (69)	493 (70)	98 (66)	323 (72)
Ever	74 (30)	205 (29)	49 (33)	122 (27)
Oral contraceptive use				
Never	93 (38)	283 (40)	58 (39)	157 (35)
Ever	150 (61)	415 (59)	90 (61)	287 (64)
Alcohol consumption				
Lifetime abstainers	109 (45)	257 (37)	43 (29)	160 (36)
Ex-drinkers	13 (5)	24 (3)	3 (2)	13 (3)
Low intake, 1–19 g/day	97 (40)	337 (48)	78 (53)	223 (50)
Medium intake, 20–39 g/day	19 (8)	61 (9)	20 (14)	38 (9)
High intake, ≥ 40 g/day	6 (2)	21 (3)	4 (3)	12 (3)
Family history of breast cancer^a^				
No	185 (76)	548 (78)	101 (68)	341 (76)
Yes	38 (16)	77 (11)	30 (20)	43 (10)
ER				
Positive	188 (77.0)		94 (63.5)	
Negative	45 (18.4)		45 (30.4)	
PR				
Positive	126 (51.6)		57 (38.5)	
Negative	106 (43.4)		80 (54.1)	
HER2				
Positive	73 (29.9)		41 (27.7)	
Negative	153 (62.7)		96 (64.9)	
Grade				
Well differentiated	62 (25.4)		24 (16.2)	
Moderately differentiated	104 (42.6)		52 (35.1)	
Poorly differentiated	65 (26.6)		61 (41.2)	
Size				
< 1 cm	76 (31.1)		25 (16.9)	
1–2 cm	116 (47.5)		51 (34.5)	
≥ 2 cm	49 (20.1)		65 (43.9)	
Nodal Status				
Positive	39 (16.0)		65 (43.9)	
Negative	190 (77.9)		71 (48.0)	

*Abbreviations*: *BMI* body mass index, *SD* standard deviation

^a^Family history of breast cancer is defined as having any relative with breast cancer

ER, PR and HER2 status was known for 95%, 94% and 93% of the cases, respectively. Within the cases with known ER, PR or HER2 status, 282 (76%) were ER+, 183 (50%) were PR+, and 114 (31%) were HER2+. Grade was known for 94% of the cases, which included 86 (23%) well differentiated, 156 (42%) moderately differentiated, and 126 (34%) poorly differentiated tumors. Lymph node involvement was known for 93% of the cases of whom 104 (28%) were node positive. Size of the tumor was known for 97% of the cases for which 101 (26%) were < 1 cm, 167 (44%) were between 1 to 2 cm and 114 (30%) were ≥ 2 cm.

Interval cases had more tumors with features predictive of poorer prognosis than screen-detected cases; ER- (30% vs 18%, *P* < 0.01), PR- (54% vs 43%, *P* = 0.02), poorly differentiated tumors (41% vs 27%, *P* < 0.01), positive nodal status (44% vs 16%, *P* < 0.001) and larger tumor size, ≥ 2 cm (44% vs 20%, *P* < 0.001).

Table 2 shows that both DA and PDA were positively associated with risk of larger breast tumors with an increase in risk of about 80% and 110% for tumors of size 2 cm and greater, respectively, per adjusted SD under all models (all test for heterogeneity by tumour size, *p* < 0.01); the risk was significant but lower for tumors of size 1–2 cm and not significant for smaller tumours of size lesser than 1 cm. DA and PDA also were positively associated with positive lymph nodes with risk gradients of about 90% and 110%, respectively, per adjusted SD under all models; whereas the risk associated to negative lymph nodes was lower (all test for heterogeneity by nodal status, p < 0.01). The model including only PDA gave the best fit when analysing tumor size (BIC = 1110, AUC = 0.68) and lymph node involvement (BIC = 1041, AUC = 0.68). BMI and NDA were not associated with the size of the tumor and nodal involvement under any model. None of the three mammographic measures and BMI were associated with the other tumor characteristics.

**Table 2:** Risk of breast cancer for BMI and mammographic measures by tumor characteristics

			OPERA (95% CI)					
		Cases/ controls	BMI + DA + NDA	BMI + PDA	BMI + DA	DA + NDA	PDA	DA
	ER							
	BIC, AUC		1130, 0.67	1117, 0.67	1130, 0.66	1108, 0.67	1094, 0.66	1107, 0.65
BMI per 1 SD at age 50	Positive	314/ 916	0.82 (0.61,1.11)	0.83 (0.61,1.12)	0.82 (0.60,1.11)			
	Negative	106/ 316	0.93 (0.62,1.41)	0.91 (0.60,1.36)	0.89 (0.59,1.35)			
BMI per 1 SD at age 70	Positive		1.23 (1.04,1.46)	1.23 (1.04,1.45)	1.23 (1.04,1.45)			
	Negative		1.12 (0.79,1.60)	1.12 (0.79,1.60)	1.11 (0.78,1.58)			
			P _for interaction with age_ = 0.07	P _for interaction with age_ = 0.08	P _for interaction with age_ = 0.06			
			*P* = 0.83	*P* = 0.88	*P* = 0.86			
DA per *adjusted* 1SD	Positive		1.32 (1.16,1.51)		1.34 (1.18,1.53)	1.33 (1.17,1.52)		1.35 (1.19,1.54)
	Negative		1.50 (1.18,1.90)		1.54 (1.21,1.95)	1.49 (1.18,1.89)		1.52 (1.21,1.92)
			*P* = 0.36		*P* = 0.33	*P* = 0.41		*P* = 0.38
PDA per *adjusted* 1SD	Positive			1.42 (1.24,1.63)			1.43 (1.25,1.64)	
	Negative			1.72 (1.33,2.23)			1.71 (1.32,2.20)	
				*P* = 0.20			*P* = 0.23	
NDA per *adjusted* 1SD	Positive		0.86 (0.76,0.98)			0.86 (0.76,0.98)		
	Negative		0.69 (0.54,0.88)			0.69 (0.54,0.88)		
			*P* = 0.10			*P* = 0.10		
	PR							
	BIC, AUC		1115, 0.68	1101, 0.68	1113, 0.67	1099, 0.67	1085, 0.66	1097, 0.65
BMI per 1 SD at age 50	Positive	206/ 604	0.77 (0.54,1.10)	0.78 (0.55,1.11)	0.77 (0.54,1.10)			
	Negative	210/ 615	0.98 (0.70,1.36)	0.96 (0.69,1.33)	0.93 (0.67,1.30)			
BMI per 1 SD at age 70	Positive		1.46 (1.18,1.81)	1.46 (1.18,1.80)	1.46 (1.18,1.80)			
	Negative		0.95 (0.76,1.19)	0.94 (0.75,1.18)	0.95 (0.75,1.18)			
			P _for interaction with age_ = 0.01	P _for interaction with age_ = 0.02	P _for interaction with age_ = 0.02			
			P = 0.02	*P* = 0.02	*P* = 0.02			
DA per *adjusted* 1SD	Positive		1.35 (1.15,1.59)		1.36 (1.16,1.60)	1.35 (1.15,1.59)		1.36 (1.16,1.60)
	Negative		1.36 (1.16,1.60)		1.43 (1.21,1.68)	1.36 (1.16,1.60)		1.42 (1.21,1.67)
			*P* = 0.96		*P* = 0.69	*P* = 0.97		*P* = 0.71
PDA per *adjusted* 1SD	Positive			1.45 (1.22,1.72)			1.45 (1.22,1.72)	
	Negative			1.54 (1.30,1.82)			1.53 (1.29,1.82)	
				*P* = 0.64			*P* = 0.64	
NDA per *adjusted* 1SD	Positive		0.87 (0.75,1.02)			0.88 (0.75,1.02)		
	Negative		0.76 (0.64,0.90)			0.76 (0.64,0.90)		
			*P* = 0.24			*P* = 0.23		
	HER2							
	BIC, AUC		1105, 0.68	1093, 0.67	1103, 0.66	1083, 0.67	1071, 0.66	1081, 0.65
BMI per 1 SD at age 50	Positive	129/ 381	0.76 (0.49,1.17)	0.78 (0.51,1.18)	0.75 (0.49,1.16)			
	Negative	281/ 824	0.92 (0.68,1.25)	0.92 (0.68,1.25)	0.90 (0.66,1.23)			
BMI per 1 SD at age 70	Positive		1.06 (0.81,1.40)	1.06 (0.81,1.40)	1.06 (0.81,1.40)			
	Negative		1.26 (1.05,1.53)	1.25 (1.04,1.51)	1.25 (1.03,1.50)			
			P _for interaction with age_ = 0.12	P _for interaction with age_ = 0.14	P _for interaction with age_ = 0.11			
			*P* = 0.41	*P* = 0.47	*P* = 0.46			
DA per *adjusted* 1SD	Positive		1.52 (1.24,1.87)		1.53 (1.24,1.89)	1.51 (1.23,1.85)		1.52 (1.24,1.87)
	Negative		1.31 (1.14,1.51)		1.36 (1.18,1.56)	1.32 (1.15,1.51)		1.36 (1.18,1.56)
			*P* = 0.25		*P* = 0.34	*P* = 0.28		*P* = 0.38
PDA per *adjusted* 1SD	Positive			1.56 (1.25,1.94)			1.55 (1.25,1.93)	
	Negative			1.47 (1.27,1.70)			1.47 (1.27,1.70)	
				*P* = 0.67			*P* = 0.69	
NDA per *adjusted* 1SD	Positive		0.97 (0.78,1.19)			0.96 (0.78,1.18)		
	Negative		0.77 (0.66,0.89)			0.77 (0.67,0.89)		
			*P* = 0.08			*P* = 0.08		
	Grade							
	BIC, AUC		1146, 0.67	1125, 0.67	1137, 0.66	1111, 0.66	1090, 0.66	1103, 0.65
BMI per 1 SD at age 50	well differentiated	96/ 271	0.93 (0.60,1.45)	0.94 (0.61,1.47)	0.94 (0.60,1.47)			
	moderately differentiated	172/ 511	0.65 (0.40,1.05)	0.66 (0.41,1.06)	0.64 (0.39,1.03)			
	poorly differentiated	146/ 436	0.94 (0.66,1.35)	0.94 (0.66,1.33)	0.93 (0.65,1.33)			
BMI per 1 SD at age 70	well differentiated		0.93 (0.60,1.45)	1.02 (0.70,1.49)	1.01 (0.69,1.48)			
	moderately differentiated		0.65 (0.40,1.05)	1.29 (1.03,1.63)	1.30 (1.04,1.64)			
	poorly differentiated		0.94 (0.66,1.35)	1.22 (0.96,1.54)	1.23 (0.98,1.56)			
			P _for interaction with age_ = 0.07	P _for interaction with age_ = 0.07	P _for interaction with age_ = 0.05			
			*P* = 0.65	*P* = 0.66	*P* = 0.58			
DA per *adjusted* 1SD	well differentiated		1.31 (1.03,1.67)		1.35 (1.06,1.72)	1.31 (1.03,1.67)		1.35 (1.06,1.72)
	moderately differentiated		1.39 (1.16,1.67)		1.42 (1.19,1.71)	1.39 (1.17,1.66)		1.43 (1.19,1.71)
	poorly differentiated		1.35 (1.12,1.64)		1.36 (1.12,1.64)	1.35 (1.11,1.64)		1.36 (1.12,1.64)
			*P* = 0.93		*P* = 0.92	*P* = 0.93		*P* = 0.91
PDA per *adjusted* 1SD	well differentiated			1.51 (1.17,1.95)			1.51 (1.17,1.95)	
	moderately differentiated			1.49 (1.23,1.80)			1.50 (1.25,1.81)	
	poorly differentiated			1.46 (1.19,1.79)			1.47 (1.20,1.80)	
				*P* = 0.98			*P* = 0.98	
NDA per *adjusted* 1SD	well differentiated		0.81 (0.63,1.04)			0.81 (0.63,1.04)		
	moderately differentiated		0.87 (0.72,1.05)			0.86 (0.71,1.04)		
	poorly differentiated		0.76 (0.63,0.91)			0.75 (0.62,0.90)		
			*P* = 0.61			*P* = 0.60		
	Size							
	BIC, AUC		1166, 0.69	1145, 0.69	1160, 0.68	1130, 0.68	1110, 0.68	1125, 0.67
BMI per 1 SD at age 50	< 1 cm	106/ 296	0.78 (0.51,1.20)	0.78 (0.51,1.21)	0.78 (0.51,1.20)			
	1–2 cm	186/ 552	0.77 (0.51,1.16)	0.78 (0.52,1.18)	0.76 (0.51,1.15)			
	≥ 2 cm	137/ 411	0.95 (0.61,1.49)	0.93 (0.60,1.45)	0.89 (0.57,1.41)			
BMI per 1 SD at age 70	< 1 cm		1.46 (1.03,2.07)	1.45 (1.02,2.06)	1.45 (1.02,2.05)			
	1–2 cm		1.15 (0.93,1.43)	1.15 (0.93,1.42)	1.15 (0.93,1.42)			
	≥ 2 cm		1.14 (0.86,1.52)	1.17 (0.88,1.55)	1.19 (0.90,1.57)			
			P _for interaction with age_ = 0.06	P _for interaction with age_ = 0.06	P _for interaction with age_ = 0.05			
			*P* = 0.74	*P* = 0.79	*P* = 0.81			
DA per *adjusted* 1SD	< 1 cm		1.00 (0.80,1.25)		1.00 (0.80,1.26)	1.00 (0.80,1.25)		1.00 (0.80,1.26)
	1–2 cm		1.32 (1.12,1.57)		1.36 (1.14,1.61)	1.32 (1.11,1.56)		1.35 (1.14,1.60)
	≥ 2 cm		1.81 (1.45,2.25)		1.83 (1.47,2.27)	1.82 (1.46,2.26)		1.84 (1.48,2.29)
			*P* < 0.01		*P* < 0.01	*P* < 0.01		*P* < 0.01
PDA per *adjusted* 1SD	< 1 cm			1.04 (0.83,1.31)			1.04 (0.83,1.30)	
	1–2 cm			1.48 (1.24,1.77)			1.48 (1.24,1.77)	
	≥ 2 cm			2.06 (1.61,2.63)			2.08 (1.63,2.65)	
				*P* < 0.01			P < 0.01	
NDA per *adjusted* 1SD	< 1 cm		0.93 (0.75,1.16)			0.95 (0.76,1.17)		
	1–2 cm		0.80 (0.67,0.95)			0.80 (0.67,0.95)		
	≥ 2 cm		0.71 (0.57,0.88)			0.70 (0.56,0.87)		
			*P* = 0.20			*P* = 0.14		
	Nodal status							
	BIC, AUC		1074, 0.69	1062, 0.69	1075, 0.68	1052, 0.68	1041, 0.68	1054, 0.67
BMI per 1 SD at age 50	Positive	116/ 341	1.09 (0.68,1.75)	1.09 (0.68,1.73)	1.02 (0.63,1.63)			
	Negative	288/ 844	0.78 (0.58,1.04)	0.78 (0.58,1.05)	0.78 (0.58,1.04)			
BMI per 1 SD at age 70	Positive		1.12 (0.81,1.53)	1.11 (0.82,1.51)	1.10 (0.81,1.49)			
	Negative		1.24 (1.04,1.49)	1.24 (1.04,1.48)	1.25 (1.04,1.49)			
			P _for interaction with age_ = 0.04	P _for interaction with age_ = 0.04	P _for interaction with age_ = 0.03			
			*P* = 0.46	*P* = 0.47	*P* = 0.56			
DA per *adjusted* 1SD	Positive		1.90 (1.49,2.43)		1.89 (1.49,2.41)	1.92 (1.51,2.46)		1.91 (1.51,2.43)
	Negative		1.24 (1.08,1.42)		1.27 (1.11,1.45)	1.24 (1.09,1.42)		1.27 (1.11,1.45)
			*P* < 0.01		*P* < 0.01	*P* < 0.01		*P* < 0.01
PDA per *adjusted* 1SD	Positive			2.11 (1.62,2.75)			2.13 (1.64,2.78)	
	Negative			1.34 (1.16,1.54)			1.34 (1.17,1.55)	
				*P* < 0.01			*P* < 0.01	
NDA per *adjusted* 1SD	Positive		0.67 (0.53,0.85)			0.68 (0.54,0.85)		
	Negative		0.86 (0.75,0.99)			0.86 (0.75,0.98)		
			*P* = 0.07			*P* = 0.09		

*Abbreviations*: *AUC* area under the receiver operating characteristic curve, *BIC* Bayesian information criterion, *BMI* body mass index, *CI* confidence interval, *DA* dense area, *NDA* non-dense area, *OPERA* Odds per Adjusted Standard Deviation, *PDA* percent dense area, *SD* standard deviation

All of the estimates from conditional logistic regression are adjusted for age at mammogram

Similar to risk of breast cancer overall, DA and PDA were positively associated with risk of screen-detected breast tumors of large size and positive lymph node involvement (Table 3). But unlike risk of breast cancer overall, models including either DA or PDA gave the best fit when analysing tumor size and lymph node involvement. For tumor size, the model including only PDA had BIC = 647 and the model including only DA had a BIC = 648 and both the models had a AUC = 0.64. For nodal status, the model including only PDA had BIC = 611 and AUC = 0.63 while the model including only DA had a BIC = 612 and AUC = 0.64. Both MD measures were associated with similar risk estimates; about 60% increase in risk of tumors of size 2 cm and greater and about 80% increase in risk of positive lymph nodes. When restricted to small tumors (< 2 cm), the positive association between MD and positive nodal involvement remained (results not shown). BMI and NDA were not associated with the size of the tumor and nodal involvement under any model. None of the three mammographic measures and BMI were associated with the other tumor characteristics for screen-detected cancer.

**Table 3:** Risk of screen-detected breast cancer for BMI and mammographic measures by tumor characteristics

			OPERA (95% CI)					
		Cases/ controls	BMI + DA + NDA	BMI + PDA	BMI + DA	DA + NDA	PDA	DA
	ER							
	BIC, AUC		659, 0.64	646, 0.64	646, 0.64	638, 0.62	625, 0.62	625, 0.62
BMI per 1 SD at age 50	Positive	188/ 537	0.95 (0.66,1.38)	0.96 (0.66,1.38)	0.96 (0.66,1.38)			
	Negative	45/ 130	1.16 (0.52,2.59)	1.14 (0.51,2.54)	1.12 (0.50,2.51)			
BMI per 1 SD at age 70	Positive		1.30 (1.05,1.61)	1.30 (1.05,1.60)	1.30 (1.05,1.61)			
	Negative		1.08 (0.65,1.80)	1.08 (0.65,1.80)	1.09 (0.65,1.82)			
			P _for interaction with age_ = 0.39	P _for interaction with age_ = 0.40	P _for interaction with age_ = 0.40			
			*P* = 0.79	*P* = 0.80	*P* = 0.82			
DA per *adjusted* 1SD	Positive		1.16 (0.99,1.36)		1.17 (0.99,1.37)	1.16 (0.99,1.37)		1.17 (0.99,1.37)
	Negative		1.30 (0.92,1.82)		1.31 (0.92,1.85)	1.29 (0.92,1.81)		1.30 (0.92,1.84)
			*P* = 0.57		*P* = 0.56	*P* = 0.59		*P* = 0.58
PDA per *adjusted* 1SD	Positive			1.17 (0.99,1.38)			1.17 (0.99,1.39)	
	Negative			1.36 (0.94,1.96)			1.35 (0.94,1.94)	
				*P* = 0.46			*P* = 0.49	
NDA per *adjusted* 1SD	Positive		0.99 (0.83,1.16)			0.99 (0.83,1.17)		
	Negative		0.87 (0.61,1.23)			0.88 (0.62,1.24)		
			*P* = 0.53			*P* = 0.55		
	PR							
	BIC, AUC		652, 0.65	640, 0.65	640, 0.65	634, 0.63	622, 0.62	622, 0.62
BMI per 1 SD at age 50	Positive	126/ 362	0.95 (0.63,1.43)	0.93 (0.62,1.40)	0.94 (0.63,1.42)			
	Negative	106/ 301	1.10 (0.62,1.97)	1.10 (0.61,1.96)	1.07 (0.60,1.90)			
BMI per 1 SD at age 70	Positive		1.48 (1.14,1.93)	1.47 (1.13,1.91)	1.48 (1.14,1.93)			
	Negative		1.01 (0.74,1.37)	1.00 (0.74,1.36)	1.01 (0.74,1.37)			
			P _for interaction with age_ = 0.23	P _for interaction with age_ = 0.21	P _for interaction with age_ = 0.22			
			*P* = 0.17	*P* = 0.17	*P* = 0.17			
DA per *adjusted* 1SD	Positive		1.21 (0.99,1.48)		1.21 (0.99,1.49)	1.19 (0.97,1.46)		1.19 (0.97,1.46)
	Negative		1.18 (0.95,1.45)		1.20 (0.97,1.48)	1.17 (0.95,1.45)		1.20 (0.97,1.48)
			*P* = 0.84		*P* = 0.94	*P* = 0.92		*P* = 0.98
PDA per *adjusted* 1SD	Positive			1.20 (0.97,1.49)			1.18 (0.96,1.46)	
	Negative			1.24 (1.00,1.54)			1.23 (0.99,1.53)	
				*P* = 0.84			*P* = 0.79	
NDA per *adjusted* 1SD	Positive		1.03 (0.84,1.27)			1.03 (0.84,1.26)		
	Negative		0.88 (0.70,1.10)			0.88 (0.70,1.11)		
			*P* = 0.30			*P* = 0.33		
	HER2							
	BIC, AUC		638, 0.66	627, 0.65	626, 0.65	619, 0.63	608, 0.63	607, 0.63
BMI per 1 SD at age 50	Positive	73/ 220	0.95 (0.56,1.63)	0.93 (0.56,1.55)	0.95 (0.56,1.60)			
	Negative	153/ 431	0.94 (0.60,1.47)	0.95 (0.60,1.49)	0.94 (0.60,1.48)			
BMI per 1 SD at age 70	Positive		0.91 (0.61,1.34)	0.92 (0.63,1.34)	0.92 (0.62,1.34)			
	Negative		1.41 (1.11,1.79)	1.40 (1.10,1.78)	1.40 (1.10,1.79)			
			P _for interaction with age_ = 0.35	P _for interaction with age_ = 0.38	P _for interaction with age_ = 0.36			
			*P* = 0.16	*P* = 0.17	*P* = 0.17			
DA per *adjusted* 1SD	Positive		1.41 (1.08,1.84)		1.40 (1.07,1.84)	1.41 (1.08,1.84)		1.41 (1.07,1.84)
	Negative		1.13 (0.94,1.35)		1.14 (0.95,1.37)	1.13 (0.94,1.35)		1.14 (0.95,1.37)
			*P* = 0.18		*P* = 0.22	*P* = 0.17		*P* = 0.20
PDA per *adjusted* 1SD	Positive			1.34 (1.02,1.77)			1.35 (1.02,1.78)	
	Negative			1.17 (0.97,1.41)			1.17 (0.97,1.41)	
				*P* = 0.41			*P* = 0.39	
NDA per *adjusted* 1SD	Positive		1.13 (0.85,1.50)			1.13 (0.85,1.49)		
	Negative		0.91 (0.75,1.11)			0.92 (0.76,1.11)		
			*P* = 0.22			*P* = 0.23		
	Grade							
	BIC, AUC		680, 0.65	660, 0.65	661, 0.65	647, 0.63	627, 0.63	628, 0.62
BMI per 1 SD at age 50	well differentiated	62/ 174	1.09 (0.66,1.79)	1.10 (0.67,1.80)	1.10 (0.67,1.80)			
	moderately differentiated	104/ 298	0.98 (0.54,1.75)	0.99 (0.55,1.77)	0.98 (0.55,1.76)			
	poorly differentiated	65/ 192	0.70 (0.34,1.44)	0.70 (0.35,1.43)	0.70 (0.34,1.44)			
BMI per 1 SD at age 70	well differentiated		1.05 (0.64,1.72)	1.04 (0.64,1.70)	1.02 (0.63,1.66)			
	moderately differentiated		1.35 (1.03,1.77)	1.34 (1.02,1.76)	1.35 (1.03,1.78)			
	poorly differentiated		1.30 (0.93,1.82)	1.30 (0.93,1.80)	1.30 (0.93,1.82)			
			P _for interaction with age_ = 0.39	P _for interaction with age_ = 0.40	P _for interaction with age_ = 0.38			
			*P* = 0.79	*P* = 0.77	*P* = 0.74			
DA per *adjusted* 1SD	well differentiated		1.29 (0.96,1.74)		1.33 (0.98,1.79)	1.29 (0.96,1.74)		1.32 (0.98,1.79)
	moderately differentiated		1.13 (0.91,1.40)		1.14 (0.92,1.41)	1.13 (0.91,1.40)		1.14 (0.92,1.41)
	poorly differentiated		1.19 (0.90,1.56)		1.19 (0.90,1.57)	1.18 (0.90,1.56)		1.18 (0.89,1.57)
			*P* = 0.77		*P* = 0.72	*P* = 0.77		*P* = 0.73
PDA per *adjusted* 1SD	well differentiated			1.40 (1.02,1.90)			1.39 (1.02,1.89)	
	moderately differentiated			1.16 (0.92,1.45)			1.17 (0.94,1.47)	
	poorly differentiated			1.16 (0.86,1.55)			1.15 (0.86,1.54)	
				*P* = 0.59			*P* = 0.62	
NDA per *adjusted* 1SD	well differentiated		0.87 (0.64,1.20)			0.88 (0.64,1.20)		
	moderately differentiated		0.97 (0.75,1.24)			0.94 (0.73,1.20)		
	poorly differentiated		1.01 (0.77,1.32)			1.01 (0.77,1.32)		
			*P* = 0.78			*P* = 0.80		
	Size							
	BIC, AUC		700, 0.67	679, 0.66	680, 0.67	667, 0.64	647, 0.64	648, 0.64
BMI per 1 SD at age 50	< 1 cm	76/ 213	0.95 (0.60,1.52)	0.96 (0.61,1.53)	0.96 (0.60,1.52)			
	1–2 cm	116/ 339	0.86 (0.49,1.51)	0.87 (0.50,1.54)	0.86 (0.49,1.52)			
	≥ 2 cm	49/ 141	0.83 (0.32,2.14)	0.83 (0.33,2.10)	0.79 (0.30,2.07)			
BMI per 1 SD at age 70	< 1 cm		1.55 (1.05,2.28)	1.54 (1.05,2.27)	1.55 (1.05,2.27)			
	1–2 cm		1.24 (0.97,1.59)	1.23 (0.96,1.58)	1.24 (0.97,1.60)			
	≥ 2 cm		1.09 (0.64,1.84)	1.10 (0.65,1.85)	1.11 (0.66,1.88)			
			P _for interaction with age_ = 0.30	P _for interaction with age_ = 0.33	P _for interaction with age_ = 0.29			
			*P* = 0.79	*P* = 0.80	*P* = 0.80			
DA per *adjusted* 1SD	< 1 cm		0.92 (0.70,1.20)		0.92 (0.70,1.20)	0.91 (0.70,1.19)		0.91 (0.70,1.19)
	1–2 cm		1.22 (0.99,1.50)		1.23 (0.99,1.52)	1.21 (0.98,1.50)		1.23 (0.99,1.52)
	≥ 2 cm		1.56 (1.11,2.19)		1.58 (1.12,2.22)	1.57 (1.12,2.20)		1.59 (1.13,2.24)
			*P* = 0.04		*P* = 0.04	*P* = 0.03		*P* = 0.03
PDA per *adjusted* 1SD	< 1 cm			0.93 (0.71,1.22)			0.92 (0.71,1.20)	
	1–2 cm			1.26 (1.01,1.57)			1.26 (1.02,1.57)	
	≥ 2 cm			1.64 (1.13,2.38)			1.66 (1.14,2.40)	
				*P* = 0.03			*P* = 0.02	
NDA per *adjusted* 1SD	< 1 cm		0.99 (0.75,1.29)			1.02 (0.78,1.33)		
	1–2 cm		0.94 (0.76,1.16)			0.93 (0.76,1.15)		
	≥ 2 cm		0.85 (0.58,1.24)			0.83 (0.57,1.21)		
			*P* = 0.81			*P* = 0.68		
	Nodal status							
	BIC, AUC		645, 0.65	632, 0.65	632, 0.65	625, 0.64	611, 0.63	612, 0.64
BMI per 1 SD at age 50	Positive	39/ 123	0.89 (0.30,2.69)	0.97 (0.33,2.84)	0.88 (0.29,2.68)			
	Negative	190/ 539	0.93 (0.65,1.32)	0.93 (0.65,1.32)	0.93 (0.65,1.33)			
BMI per 1 SD at age 70	Positive		1.61 (1.04,2.50)	1.56 (1.01,2.42)	1.61 (1.03,2.51)			
	Negative		1.17 (0.94,1.46)	1.16 (0.93,1.45)	1.17 (0.94,1.46)			
			P _for interaction with age_ = 0.39	P _for interaction with age_ = 0.46	P _for interaction with age_ = 0.38			
			*P* = 0.41	*P* = 0.45	*P* = 0.43			
DA per *adjusted* 1SD	Positive		1.79 (1.22,2.62)		1.78 (1.21,2.61)	1.76 (1.22,2.56)		1.78 (1.22,2.59)
	Negative		1.11 (0.94,1.30)		1.12 (0.95,1.31)	1.11 (0.94,1.30)		1.11 (0.95,1.31)
			*P* = 0.02		*P* = 0.02	*P* = 0.02		*P* = 0.02
PDA per *adjusted* 1SD	Positive			1.85 (1.22,2.79)			1.84 (1.23,2.76)	
	Negative			1.13 (0.96,1.34)			1.13 (0.96,1.34)	
				*P* = 0.02			*P* = 0.02	
NDA per *adjusted* 1SD	Positive		0.86 (0.59,1.27)			0.87 (0.59,1.29)		
	Negative		0.96 (0.81,1.13)			0.95 (0.81,1.13)		
			*P* = 0.63			*P* = 0.67		

*Abbreviations*: *AUC* area under the receiver operating characteristic curve, *BIC* Bayesian information criterion, *BMI* body mass index, *CI* confidence interval, *DA* dense area, *NDA* non-dense area, *OPERA* Odds per Adjusted Standard Deviation, *PDA* percent dense area, *SD* standard deviation

All of the estimates from conditional logistic regression are adjusted for age at mammogram

The association between risk of interval cancer and DA, NDA and PDA did not vary by any of the tumor characteristics (Table 4). Higher BMI was associated with a decreased risk of negative lymph nodes at 50 years and increased risk of negative lymph nodes at 70 years.

**Table 4:** Risk of interval breast cancer for BMI and mammographic measures by tumor characteristics

			OPERA (95% CI)					
		Cases/ controls	BMI + DA + NDA	BMI + PDA	BMI + DA	DA + NDA	PDA	DA
	ER							
	BIC, AUC		373, 0.77	363, 0.77	380, 0.73	349, 0.77	339, 0.77	356, 0.73
BMI per 1 SD at age 50	Positive	94/ 278	0.71 (0.38,1.33)	0.70 (0.38,1.31)	0.74 (0.40,1.35)			
	Negative	45/ 145	0.91 (0.51,1.61)	0.88 (0.50,1.56)	0.90 (0.51,1.61)			
BMI per 1 SD at age 70	Positive		1.04 (0.71,1.51)	1.03 (0.71,1.50)	0.98 (0.68,1.42)			
	Negative		1.05 (0.49,2.25)	0.96 (0.45,2.03)	0.93 (0.44,1.97)			
			P _for interaction with age_ = 0.65	P _for interaction with age_ = 0.66	P _for interaction with age_ = 0.79			
			*P* = 0.82	*P* = 0.87	*P* = 0.90			
DA per *adjusted* 1SD	Positive		2.14 (1.59,2.89)		2.12 (1.58,2.85)	2.13 (1.58,2.86)		2.10 (1.57,2.82)
	Negative		1.84 (1.20,2.83)		1.90 (1.27,2.84)	1.82 (1.20,2.73)		1.85 (1.27,2.71)
			*P* = 0.58		*P* = 0.67	*P* = 0.54		*P* = 0.60
PDA per *adjusted* 1SD	Positive			2.63 (1.87,3.69)			2.60 (1.85,3.64)	
	Negative			2.34 (1.46,3.76)			2.26 (1.44,3.56)	
				*P* = 0.70			*P* = 0.64	
NDA per *adjusted* 1SD	Positive		0.64 (0.49,0.85)			0.65 (0.49,0.85)		
	Negative		0.53 (0.35,0.81)			0.53 (0.35,0.81)		
			*P* = 0.46			*P* = 0.44		
	PR							
	BIC, AUC		362, 0.78	352, 0.78	370, 0.74	344, 0.77	334, 0.77	352, 0.73
BMI per 1 SD at age 50	Positive	57/ 172	0.56 (0.23,1.33)	0.56 (0.24,1.32)	0.59 (0.26,1.36)			
	Negative	80/ 245	0.94 (0.58,1.53)	0.91 (0.57,1.46)	0.91 (0.56,1.48)			
BMI per 1 SD at age 70	Positive		1.62 (0.94,2.80)	1.54 (0.91,2.62)	1.43 (0.87,2.36)			
	Negative		0.64 (0.39,1.04)	0.63 (0.39,1.03)	0.63 (0.38,1.02)			
			P _for interaction with age_ = 0.13	P _for interaction with age_ = 0.16	P _for interaction with age_ = 0.20			
			*P* = 0.04	*P* = 0.05	*P* = 0.06			
DA per *adjusted* 1SD	Positive		2.32 (1.56,3.44)		2.10 (1.46,3.01)	2.32 (1.58,3.41)		2.11 (1.48,3.01)
	Negative		1.91 (1.36,2.69)		2.04 (1.46,2.83)	1.82 (1.32,2.52)		1.92 (1.41,2.62)
			*P* = 0.46		*P* = 0.91	*P* = 0.35		*P* = 0.70
PDA per *adjusted* 1SD	Positive			2.92 (1.84,4.65)			2.89 (1.84,4.56)	
	Negative			2.41 (1.67,3.49)			2.29 (1.61,3.25)	
				*P* = 0.52			*P* = 0.42	
NDA per *adjusted* 1SD	Positive		0.58 (0.40,0.83)			0.60 (0.43,0.86)		
	Negative		0.59 (0.43,0.82)			0.59 (0.43,0.81)		
			*P* = 0.93			*P* = 0.91		
	HER2							
	BIC, AUC		368, 0.77	361, 0.76	375, 0.73	345, 0.76	337, 0.76	351, 0.73
BMI per 1 SD at age 50	Positive	41/ 123	0.66 (0.28,1.56)	0.65 (0.28,1.54)	0.67 (0.28,1.58)			
	Negative	96/ 293	0.91 (0.57,1.47)	0.89 (0.56,1.43)	0.92 (0.58,1.47)			
BMI per 1 SD at age 70	Positive		0.91 (0.47,1.77)	0.93 (0.49,1.77)	0.87 (0.45,1.70)			
	Negative		1.08 (0.73,1.59)	1.06 (0.72,1.55)	1.01 (0.70,1.48)			
			P _for interaction with age_ = 0.78	P _for interaction with age_ = 0.77	P _for interaction with age_ = 0.88			
			*P* = 0.62	*P* = 0.68	*P* = 0.66			
DA per *adjusted* 1SD	Positive		2.15 (1.39,3.30)		2.19 (1.42,3.38)	2.03 (1.34,3.07)		2.05 (1.36,3.11)
	Negative		1.96 (1.47,2.63)		1.95 (1.48,2.58)	1.95 (1.46,2.60)		1.94 (1.48,2.56)
			*P* = 0.74		*P* = 0.66	*P* = 0.88		*P* = 0.83
PDA per *adjusted* 1SD	Positive			2.43 (1.50,3.95)			2.29 (1.44,3.66)	
	Negative			2.43 (1.76,3.36)			2.41 (1.75,3.31)	
				*P* = 1.00			P = 0.87	
NDA per *adjusted* 1SD	Positive		0.82 (0.54,1.24)			0.82 (0.55,1.22)		
	Negative		0.55 (0.41,0.73)			0.55 (0.41,0.74)		
			*P* = 0.13			*P* = 0.12		
	Grade							
	BIC, AUC		386, 0.78	375, 0.77	387, 0.75	354, 0.77	343, 0.76	356, 0.74
BMI per 1 SD at age 50	well differentiated	24/ 66	0.62 (0.21,1.82)	0.60 (0.20,1.77)	0.67 (0.24,1.91)			
	moderately differentiated	52/ 165	0.40 (0.13,1.22)	0.38 (0.13,1.13)	0.39 (0.13,1.18)			
	poorly differentiated	61/ 187	1.01 (0.62,1.66)	0.98 (0.61,1.58)	0.99 (0.62,1.59)			
BMI per 1 SD at age 70	well differentiated		0.64 (0.24,1.67)	0.61 (0.22,1.64)	0.63 (0.24,1.65)			
	moderately differentiated		1.18 (0.66,2.11)	1.23 (0.70,2.17)	1.08 (0.61,1.92)			
	poorly differentiated		1.08 (0.67,1.74)	1.14 (0.73,1.79)	1.22 (0.80,1.86)			
			P _for interaction with age_ = 0.53	P _for interaction with age_ = 0.41	P _for interaction with age_ = 0.52			
			*P* = 0.36	*P* = 0.28	*P* = 0.24			
DA per *adjusted* 1SD	well differentiated		1.61 (0.97,2.70)		1.64 (0.97,2.77)	1.49 (0.92,2.41)		1.49 (0.92,2.43)
	moderately differentiated		2.64 (1.70,4.11)		2.77 (1.77,4.33)	2.66 (1.71,4.13)		2.75 (1.76,4.29)
	poorly differentiated		1.85 (1.28,2.68)		1.77 (1.28,2.45)	1.86 (1.29,2.68)		1.80 (1.31,2.47)
			*P* = 0.29		*P* = 0.19	*P* = 0.20		*P* = 0.14
PDA per *adjusted* 1SD	well differentiated			2.11 (1.17,3.80)			1.90 (1.09,3.32)	
	moderately differentiated			2.94 (1.83,4.71)			2.92 (1.82,4.69)	
	poorly differentiated			2.24 (1.51,3.33)			2.27 (1.54,3.34)	
				*P* = 0.60			*P* = 0.49	
NDA per *adjusted* 1SD	well differentiated		0.78 (0.48,1.26)			0.80 (0.50,1.28)		
	moderately differentiated		0.73 (0.48,1.10)			0.72 (0.49,1.06)		
	poorly differentiated		0.48 (0.33,0.71)			0.48 (0.33,0.70)		
			*P* = 0.20			*P* = 0.16		
	Size							
	BIC, AUC		395, 0.78	381, 0.78	397, 0.75	359, 0.78	345, 0.77	361, 0.74
BMI per 1 SD at age 50	< 1 cm	25/ 70	0.71 (0.25,2.00)	0.70 (0.25,1.99)	0.69 (0.23,2.09)			
	1–2 cm	51/ 154	0.69 (0.31,1.54)	0.70 (0.32,1.52)	0.73 (0.35,1.52)			
	≥ 2 cm	65/ 204	0.99 (0.52,1.88)	0.96 (0.51,1.78)	0.99 (0.52,1.89)			
BMI per 1 SD at age 70	< 1 cm		0.80 (0.23,2.85)	0.81 (0.23,2.89)	0.82 (0.23,2.92)			
	1–2 cm		1.03 (0.60,1.76)	0.96 (0.56,1.62)	0.91 (0.54,1.55)			
	≥ 2 cm		0.92 (0.54,1.57)	0.94 (0.55,1.59)	0.94 (0.58,1.53)			
			P _for interaction with age_ = 0.90	P _for interaction with age_ = 0.95	P _for interaction with age_ = 0.97			
			*P* = 0.93	*P* = 0.94	*P* = 0.93			
DA per *adjusted* 1SD	< 1 cm		1.62 (0.92,2.83)		1.62 (0.92,2.87)	1.57 (0.91,2.70)		1.56 (0.91,2.69)
	1–2 cm		1.65 (1.14,2.38)		1.65 (1.16,2.33)	1.61 (1.13,2.31)		1.62 (1.15,2.28)
	≥ 2 cm		2.78 (1.86,4.15)		2.74 (1.87,4.01)	2.74 (1.85,4.06)		2.71 (1.87,3.93)
			*P* = 0.11		*P* = 0.11	*P* = 0.09		*P* = 0.08
PDA per *adjusted* 1SD	< 1 cm			1.76 (0.99,3.15)			1.71 (0.97,2.99)	
	1–2 cm			2.09 (1.40,3.12)			2.05 (1.38,3.03)	
	≥ 2 cm			3.50 (2.20,5.58)			3.44 (2.19,5.41)	
				*P* = 0.12			*P* = 0.10	
NDA per *adjusted* 1SD	< 1 cm		0.83 (0.53,1.29)			0.83 (0.54,1.27)		
	1–2 cm		0.54 (0.37,0.79)			0.54 (0.37,0.79)		
	≥ 2 cm		0.58 (0.40,0.83)			0.58 (0.40,0.83)		
			*P* = 0.32			*P* = 0.32		
	Nodal status							
	BIC, AUC		354, 0.80	345, 0.79	362, 0.76	339, 0.78	331, 0.77	347, 0.74
BMI per 1 SD at age 50	Positive	65/ 189	1.34 (0.73,2.47)	1.32 (0.72,2.41)	1.31 (0.71,2.42)			
	Negative	71/ 224	0.46 (0.23,0.93)	0.45 (0.22,0.91)	0.48 (0.24,0.96)			
BMI per 1 SD at age 70	Positive		0.64 (0.36,1.14)	0.65 (0.37,1.12)	0.62 (0.35,1.09)			
	Negative		1.61 (1.04,2.49)	1.60 (1.03,2.49)	1.58 (1.03,2.41)			
			P _for interaction with age_ = 0.01	P _for interaction with age_ = 0.01	P _for interaction with age_ = 0.01			
			*P* = 0.01	*P* = 0.01	*P* = 0.01			
DA per *adjusted* 1SD	Positive		2.32 (1.57,3.41)		2.30 (1.60,3.31)	2.23 (1.55,3.23)		2.20 (1.56,3.09)
	Negative		1.92 (1.39,2.66)		1.92 (1.40,2.64)	1.84 (1.35,2.52)		1.85 (1.36,2.51)
			*P* = 0.47		*P* = 0.46	*P* = 0.43		*P* = 0.46
PDA per *adjusted* 1SD	Positive			2.69 (1.79,4.07)			2.61 (1.76,3.88)	
	Negative			2.47 (1.69,3.61)			2.32 (1.62,3.33)	
				*P* = 0.76			*P* = 0.66	
NDA per *adjusted* 1SD	Positive		0.58 (0.41,0.81)			0.57 (0.41,0.80)		
	Negative		0.60 (0.43,0.84)			0.62 (0.45,0.85)		
			*P* = 0.87			*P* = 0.72		

*Abbreviations*: *AUC* area under the receiver operating characteristic curve, *BIC* Bayesian information criterion, *BMI* body mass index, *CI* confidence interval, *DA* dense area, *NDA* non-dense area, *OPERA* Odds per Adjusted Standard Deviation, *PDA* percent dense area, *SD* standard deviation

All of the estimates from conditional logistic regression are adjusted for age at mammogram

None of the three mammographic measures were associated with risk of interval versus screen-detected breast cancer by any of the tumor characteristics (Table 5). BMI was negatively associated with risk of interval versus screen-detected cancer for tumors of 1 to 2 cm in size but not after adjusting for the mammographic measures. BMI was, however, associated with decreased risk of interval cancer with positive lymph nodes compared to screen-detected cancer, both significantly and marginally significantly after adjusting for the mammographic measures (all test for heterogeneity by nodal status, *p* = 0.02, 0.03 and 0.06).

**Table 5:** Risk of interval versus screen-detected breast cancer for BMI and mammographic measures by tumor charactersitics

			OPERA (95% CI)					
		Interval/ screen-detected cases	BMI + DA + NDA	BMI + PDA	BMI + DA	DA + NDA	PDA	DA
	ER							
	BIC, AUC		492, 0.70	481, 0.70	492, 0.67	484, 0.69	474, 0.69	485, 0.66
BMI per 1 SD	Positive	94/ 188	0.81 (0.63,1.05)	0.80 (0.62,1.04)	0.79 (0.60,1.02)			
	Negative	45/ 45	0.77 (0.49,1.21)	0.79 (0.50,1.24)	0.79 (0.50,1.24)			
			*P* = 0.82	*P* = 0.95	*P* = 0.98			
DA per *adjusted* 1SD	Positive		1.55 (1.20,2.02)		1.58 (1.21,2.04)	1.54 (1.19,1.99)		1.55 (1.20,2.01)
	Negative		1.78 (1.12,2.82)		1.90 (1.20,3.00)	1.78 (1.12,2.84)		1.88 (1.19,2.97)
			*P* = 0.61		*P* = 0.47	*P* = 0.58		*P* = 0.46
PDA per *adjusted* 1SD	Positive			1.87 (1.41,2.48)			1.86 (1.41,2.47)	
	Negative			2.22 (1.37,3.59)			2.21 (1.36,3.59)	
				*P* = 0.52			*P* = 0.53	
NDA per *adjusted* 1SD	Positive		0.68 (0.53,0.89)			0.67 (0.52,0.87)		
	Negative		0.63 (0.40,0.98)			0.64 (0.41,1.00)		
			*P* = 0.75			*P* = 0.85		
	PR							
	BIC, AUC		488, 0.70	477, 0.70	488, 0.67	480, 0.69	470, 0.69	481, 0.66
BMI per 1 SD	Positive	57/ 126	0.75 (0.55,1.03)	0.74 (0.54,1.02)	0.74 (0.53,1.02)			
	Negative	80/ 106	0.84 (0.61,1.16)	0.85 (0.61,1.17)	0.82 (0.60,1.14)			
			*P* = 0.64	*P* = 0.56	*P* = 0.63			
DA per *adjusted* 1SD	Positive		1.65 (1.20,2.29)		1.67 (1.21,2.30)	1.59 (1.16,2.18)		1.60 (1.17,2.19)
	Negative		1.59 (1.15,2.20)		1.63 (1.18,2.25)	1.60 (1.16,2.22)		1.64 (1.19,2.27)
			*P* = 0.86		*P* = 0.92	*P* = 0.97		*P* = 0.91
PDA per *adjusted* 1SD	Positive			2.01 (1.42,2.85)			1.93 (1.38,2.71)	
	Negative			1.91 (1.34,2.70)			1.94 (1.37,2.76)	
				*P* = 0.82			*P* = 0.98	
NDA per *adjusted* 1SD	Positive		0.64 (0.47,0.87)			0.64 (0.47,0.87)		
	Negative		0.71 (0.51,0.99)			0.71 (0.51,0.98)		
			*P* = 0.61			*P* = 0.66		
	HER2							
	BIC, AUC		485, 0.70	478, 0.69	488, 0.66	475, 0.69	468, 0.68	479, 0.65
BMI per 1 SD	Positive	41/ 73	0.93 (0.61,1.42)	0.92 (0.61,1.37)	0.88 (0.59,1.32)			
	Negative	96/ 153	0.84 (0.64,1.10)	0.85 (0.65,1.12)	0.84 (0.64,1.10)			
			*P* = 0.70	*P* = 0.76	*P* = 0.84			
DA per *adjusted* 1SD	Positive		1.59 (1.06,2.40)		1.61 (1.08,2.40)	1.58 (1.06,2.35)		1.58 (1.07,2.34)
	Negative		1.51 (1.14,1.99)		1.58 (1.20,2.08)	1.51 (1.15,1.99)		1.58 (1.20,2.08)
			*P* = 0.82		*P* = 0.95	*P* = 0.85		*P* = 1.00
PDA per *adjusted* 1SD	Positive			1.75 (1.14,2.69)			1.75 (1.14,2.68)	
	Negative			1.92 (1.43,2.57)			1.92 (1.43,2.58)	
				*P* = 0.72			*P* = 0.71	
NDA per *adjusted* 1SD	Positive		0.80 (0.50,1.30)			0.79 (0.49,1.25)		
	Negative		0.58 (0.44,0.78)			0.58 (0.44,0.78)		
			*P* = 0.26			*P* = 0.29		
	Grade							
	BIC, AUC		497, 0.72	486, 0.70	495, 0.68	484, 0.71	473, 0.69	483, 0.67
BMI per 1 SD	well differentiated	24/ 62	0.88 (0.55,1.42)	0.87 (0.55,1.38)	0.87 (0.54,1.38)			
	moderately differentiated	52/ 104	0.67 (0.45,0.98)	0.68 (0.46,0.99)	0.65 (0.44,0.96)			
	poorly differentiated	61/ 65	0.95 (0.66,1.35)	1.00 (0.71,1.40)	1.04 (0.75,1.44)			
			*P* = 0.39	*P* = 0.31	*P* = 0.19			
DA per *adjusted* 1SD	well differentiated		1.12 (0.69,1.84)		1.13 (0.69,1.83)	1.11 (0.68,1.81)		1.11 (0.69,1.80)
	moderately differentiated		1.97 (1.36,2.84)		1.99 (1.38,2.87)	1.92 (1.34,2.74)		1.93 (1.36,2.76)
	poorly differentiated		1.52 (1.05,2.19)		1.60 (1.12,2.28)	1.52 (1.05,2.20)		1.62 (1.14,2.30)
			*P* = 0.18		*P* = 0.18	*P* = 0.19		*P* = 0.18
PDA per *adjusted* 1SD	well differentiated			1.29 (0.79,2.11)			1.29 (0.79,2.11)	
	moderately differentiated			2.16 (1.47,3.18)			2.13 (1.46,3.10)	
	poorly differentiated			2.02 (1.37,2.99)			2.04 (1.38,3.01)	
				*P* = 0.23			*P* = 0.24	
NDA per *adjusted* 1SD	well differentiated		0.86 (0.55,1.33)			0.84 (0.54,1.30)		
	moderately differentiated		0.82 (0.56,1.20)			0.81 (0.56,1.16)		
	poorly differentiated		0.45 (0.30,0.70)			0.45 (0.29,0.70)		
			*P* = 0.06			*P* = 0.06		
	Size							
	BIC, AUC		513, 0.72	498, 0.72	509, 0.69	500, 0.71	485, 0.71	497, 0.68
BMI per 1 SD	< 1 cm	25/ 76	0.74 (0.47,1.16)	0.74 (0.47,1.16)	0.71 (0.45,1.12)			
	1–2 cm	51/ 116	0.71 (0.48,1.05)	0.70 (0.48,1.04)	0.68 (0.46,1.00)			
	≥ 2 cm	65/ 49	0.95 (0.64,1.40)	0.97 (0.66,1.42)	1.01 (0.71,1.46)			
			*P* = 0.54	*P* = 0.47	*P* = 0.27			
DA per *adjusted* 1SD	< 1 cm		1.49 (0.92,2.42)		1.55 (0.97,2.50)	1.52 (0.94,2.46)		1.58 (0.99,2.54)
	1–2 cm		1.26 (0.88,1.80)		1.27 (0.89,1.82)	1.29 (0.91,1.83)		1.31 (0.92,1.86)
	≥ 2 cm		2.02 (1.39,2.94)		2.12 (1.46,3.07)	2.04 (1.41,2.95)		2.16 (1.50,3.10)
			*P* = 0.16		*P* = 0.12	*P* = 0.18		*P* = 0.12
PDA per *adjusted* 1SD	< 1 cm			1.75 (1.08,2.82)			1.81 (1.12,2.93)	
	1–2 cm			1.48 (1.01,2.15)			1.54 (1.07,2.22)	
	≥ 2 cm			2.67 (1.77,4.03)			2.70 (1.80,4.06)	
				*P* = 0.07			*P* = 0.09	
NDA per *adjusted* 1SD	< 1 cm		0.75 (0.50,1.13)			0.73 (0.49,1.10)		
	1–2 cm		0.74 (0.53,1.02)			0.72 (0.52,0.99)		
	≥ 2 cm		0.49 (0.30,0.81)			0.49 (0.30,0.81)		
			*P* = 0.33			*P* = 0.37		
	Nodal Status							
	BIC, AUC		481, 0.72	475, 0.71	487, 0.68	475, 0.71	469, 0.69	480, 0.66
BMI per 1 SD	Positive	65/ 39	0.58 (0.38,0.90)	0.61 (0.41,0.93)	0.64 (0.42,0.97)			
	Negative	71/ 190	1.04 (0.81,1.35)	1.04 (0.81,1.35)	1.02 (0.79,1.32)			
			*P* = 0.02	*P* = 0.03	*P* = 0.06			
DA per *adjusted* 1SD	Positive		1.75 (1.18,2.58)		1.97 (1.33,2.91)	1.60 (1.09,2.36)		1.77 (1.22,2.55)
	Negative		1.48 (1.12,1.95)		1.49 (1.13,1.96)	1.47 (1.12,1.94)		1.48 (1.13,1.95)
			*P* = 0.48		*P* = 0.23	*P* = 0.71		*P* = 0.43
PDA per *adjusted* 1SD	Positive			2.47 (1.63,3.74)			2.19 (1.46,3.28)	
	Negative			1.71 (1.28,2.30)			1.70 (1.27,2.28)	
				*P* = 0.13			*P* = 0.29	
NDA per *adjusted* 1SD	Positive		0.44 (0.27,0.70)			0.45 (0.28,0.74)		
	Negative		0.73 (0.56,0.95)			0.74 (0.57,0.96)		
			*P* = 0.05			*P* = 0.07		

*Abbreviations*: *AUC* area under the receiver operating characteristic curve, *BIC* Bayesian information criterion, *BMI* body mass index, *CI* confidence interval, *DA* dense area, *NDA* non-dense area, *OPERA* Odds per Adjusted Standard Deviation, *PDA* percent dense area, *SD* standard deviation

All of the estimates from conditional logistic regression are adjusted for age at mammogram

The findings above were similar when we adjusted for all the confounders. No substantial differences in estimates were observed from the sensitivity analyses.

## Discussion

As assumed in our previous paper [6], that risk of screen-detected cancers mostly reflects inherent cancer risk, and the predictors of interval versus screen-detected disease mostly reflect predictors of masking, we found that both DA and PDA are positively associated with inherent risk of larger breast tumors and positive nodal involvement. Associations between DA, NDA and PDA, and risk of masking of tumors did not vary by tumor characteristics. None of the three mammographic measures were associated with differential nodal involvement for interval versus screen-detected cancer. However, BMI was associated with a decreased risk of positive nodal involvement for interval versus screen-detected cancer.

Our results are similar to those of a large study consisting of six pooled datasets, which also found DA and PDA to be associated with increased risk of larger tumors, but found only PDA to be positively associated with positive nodal status [18]. Unlike our study, the pooled study [18] had also found DA to be positively associated with ER+ and PR+ tumors, and NDA to be negatively associated with the size of the tumor and HER2+. A meta-analysis [19], however, found no differential association between MD and risk by ER and HER2 status of tumors. The differences in the results could be due to the fact that the previous reports did not allow the relation between relative risk and BMI to depend on age.

Some studies analysing screen-detected breast cancer had also found MD to be associated with increased risk of larger tumors [7, 10] and positive nodal involvement [7], and none of them allowed the relation between risk and BMI to depend on age. Only one study, which did not adjust for BMI, found no association between MD and size of the tumor or nodal status for screen-detected breast cancer [9]. We had interpreted risk of screen-detected breast cancer to be mainly representative of risk of developing a detectable breast tumor, assuming that the cases did not have a detectable tumor at prior mammograms. As mentioned in our previous paper [6], this assumption could be reasonable based on a review [20] which found that within interval cases, which consists of true interval cases, false-negative cases (tumor is not identified at a mammogram due to reader error) and occult tumors (tumor is not identified at a mammogram due to high density), there was a lesser percentage of the latter two cases; false-negative cases (25–40%) and occult tumors (8–12%). Our findings, therefore, suggest there is a biological relationship between the amount of dense tissues in the breast and faster growth rate of the tumor. When restricted to small tumors (<2 cm), the positive association between MD and positive nodal involvement remained which could indicate that besides faster growth rate, there might be another biological mechanism that is involved between dense tissues and spreading of the tumor to the lymph nodes. Overall, this would support the indication that more frequent screening schedules should be offered to women with greater age- and BMI-adjusted MD.

We found no association between any of the mammographic measures and risk of interval breast cancer by tumor characteristics. Few studies had investigated risk by tumor characteristics separately for interval cancer. One study found greater MD to be associated with increased risk of ER+ interval cancer but it had not adjusted for BMI [9]. Another study found an inverse association between MD and grade of tumor for interval cancers, but it had only allowed the association between risk and BMI to be a constant. To our knowledge, none of the studies had reported on the association between BMI and risk of interval cancer by tumor characteristics after adjusting for the mammographic measures. However, our finding of BMI being associated with increased risk of interval cancer with positive lymph nodes at age 50 is consistent with earlier studies that had analysed cases overall [21, 22], while another study found no correlation between the number of lymph nodes affected and BMI [23]. None of these studies had allowed the association with BMI to vary by age and the mean age of their study participants was younger, between 47 to 50 years. As mentioned in our previous study [6], risk of interval cancer is more likely a combination of risk of developing the tumor and risk of masking, and also influenced by the rate of tumor growth. Consequently, the results for risk of interval cancer are difficult to interpret.

We found no association between all three mammographic measures and risk of interval versus screen-detected cancer by tumor characteristics. A study which had further categorised interval cancer into true interval, false negatives, minimal-sign cancers and occult tumors also found no association between PDA and risk of each of the interval cancer category mentioned above compared with screen-detected cancer for the tumor phenotype, luminal A, luminal B, HER2 and triple-negative [24]. Another study, which had stratified by PDA (low versus high), found that for women with low PDA, interval cancers were more likely to be HER2+ and have positive nodal involvement compared with screen-detected cancers [25]. Both the studies, however, did not adjust for BMI [24, 25]. Our results for BMI, however, needs to be confirmed by other studies as to our knowledge this is the first study to investigate the association between BMI and risk by tumor characteristic and taking into account the detection mode.

To our knowledge, our study is the first to estimate the differential risk of developing breast cancer and risk of masking by tumor characteristics through investigating the concurrent associations with all three mammographic measures, DA, NDA and PDA stratified by detection mode. BMI has been measured at cohort entry and we had realistically allowed the BMI association to vary by age since BMI is known to have differential associations on breast cancer risk; BMI is negatively associated with breast cancer risk for premenopausal women and positively associated with breast cancer risk for postmenopausal women [26]. A limitation of our study is the sample size, especially for small subgroups. Our ER, PR and HER2 status of tumors was measured by only one pathologist using immunohistochemistry method which might lead to non-differential misclassification of the status of the tumors and this most likely will attenuate the risk estimates towards null. Other limitations have been mentioned in our previous paper [6].

## Conclusions

We found that both DA and PDA predicted inherent risk of larger breast tumors and positive nodal status. There was no differential risk of masking by tumor characteristics associated with DA, NDA and PDA. None of the three mammographic measures were associated with differential nodal involvement for interval versus screen-detected cancer. Our finding suggest that the dense tissues of the breast play a role in faster growth rate of the tumor and in spreading of the tumor to the lymph nodes that would not be explained by delay of the diagnosis due to masking.

## Abbreviations

AUC: Area under the receiver operating characteristic curve
BIC: Bayesian information criterion
BMI: Body mass index
DA: Dense area
MCCS: Melbourne Collaborative Cohort Study
MD: Mammographic density
NDA: Non-dense area
OPERA: Odds per Adjusted Standard Deviation
PDA: Percent dense area
SD: Standard deviation
VCR: Victorian Cancer Registry
